# Outcomes of image-based sexual abuse among young people: a systematic review

**DOI:** 10.3389/fpsyg.2025.1599087

**Published:** 2025-07-10

**Authors:** Per Moum Hellevik, Linn-Eirin Aronsen Haugen, Carolina Överlien

**Affiliations:** ^1^Norwegian Centre for Violence and Traumatic Stress Studies, Oslo, Norway; ^2^Uppsala University, National Centre for Knowledge on Men's Violence against Women (NCK), Uppsala, Sweden

**Keywords:** image-based sexual abuse, digital victimization, forensic psychology, adolescent mental health, victim blaming, gender-based violence

## Abstract

Image-Based Sexual Abuse (IBSA) is an increasingly recognized issue, especially among young people, yet empirical research on its consequences remains relatively scarce. Following PRISMA guidelines, a systematic search identified peer-reviewed studies published between 2010 and 2024, and eligible studies were screened and quality-assessed using standardized criteria. This systematic review synthesizes findings from 12 empirical studies to provide a comprehensive overview of the psychological, social, educational and occupational consequences of IBSA victimization among individuals aged 10–24. The findings highlight severe emotional and psychological distress, including fear, anxiety, depression, and suicidal ideation. Social repercussions such as bullying, ostracization, and victim-blaming further exacerbate these impacts. Additionally, IBSA is associated with significant disruptions in educational and occupational trajectories, with victims reporting school relocation and/or job loss. These outcomes underscore the parallels between IBSA and physical sexual abuse, emphasizing the need for targeted prevention strategies, improved legal frameworks, and informed victim support services. The study calls for further research into the long-term consequences of IBSA and the development of interventions that address both its digital and societal dimensions. Given the legal and psychological severity of IBSA, this review also highlights the need for forensic psychological assessment, integration of victim experiences into legal processes, and the development of trauma-informed policies that support young people through the justice system.

## 1 Introduction

The use of digital technology in facilitating violent and abusive behavior has received increasing attention over several years (Mitchell et al., 2022). One form of such victimization, image-based sexual abuse (IBSA), has frequently been highlighted in research on sexting, an activity defined as “… the sending or receiving of sexually explicit pictures, videos, or text messages via smartphone, digital camera, or computer” (Strasburger et al., 2019, p. 1). However, as this definition illustrates, sexting does not necessarily indicate abuse or victimization. Conflating sexting with IBSA is therefore unfortunate, as people engaging in sexting in many instances view this as a voluntary, positive and intimate activity (Barrense-Dias et al., 2017, 2019; Ringrose et al., 2022a). Contrary, IBSA, understood as non-consensual taking, sharing, and/or threats to share nude or sexual images (Henry et al., 2020), is strictly harmful and abusive behavior. While more data are needed to describe the prevalence of such abuse accurately, studies indicate that many young people have experienced IBSA victimization (Henry et al., 2020; OeSC, 2017; Ruvalcaba and Eaton, 2019; Barroso et al., 2021). Crucially, such victimization appears to have several negative impacts on those involved, and research on IBSA in relation to sexting has found that victims experience significant harm, including psychological distress, social isolation, and even loss of educational opportunities or employment (Doyle et al., 2021). IBSA also raises critical concerns at the intersection of psychology and law. The psychological impacts reported by young victims often mirror those found in victims of offline sexual abuse (Henry et al., 2020), yet legal systems have historically struggled to address technology-facilitated forms of violence (McGlynn et al., 2019). Forensic psychologists can play a crucial role in the assessment of trauma, risk, and credibility in legal contexts, especially when victim-blaming or disbelief is present (Anderson and Overby, 2021). Understanding IBSA within a forensic framework is essential for guiding appropriate legal responses, improving investigative interviewing, and enhancing support in judicial settings. Despite the growing recognition of IBSA, much is unknown about its outcomes. While studies have looked at factors associated with IBSA victimization, such as substance abuse, risky sexual behavior (i.e. behaviors increasing the risk of STIs or unplanned pregnancies) and other forms of violence victimization and perpetration (Pedersen et al., 2023; Sparks et al., 2023; Barroso et al., 2021), fewer have looked at outcomes directly related to such abuse. This paper will present a systematic review of the available research on the outcomes of IBSA among young people. Specifically, we will examine the psychological/emotional, social, educational and occupational consequences experienced by victims of such abuse. By synthesizing the available evidence, we aim to provide a comprehensive overview of the harm caused by IBSA.

## 2 Current research on outcomes of sexting

While few studies have looked specifically at the potential outcomes of IBSA, more is known about the outcomes of sexting. To our knowledge, three systematic reviews have summarized relevant research on the outcomes of sexting (Anastassiou, 2017; Dodaj et al., 2022; Doyle et al., 2021).

Anastassiou (2017) reviewed eight qualitative papers on the topic of sexting in order to identify how sexting affects young people's wellbeing. The review found that for many young people, sexting was seen as a fun and positive activity. Furthermore, sexting was, in some instances, seen as part of non-physical sexual exploration. However, Anastassiou (2017) also found that sexting could negatively impact young people through reputational damage, where having intimate images shared non-consensually resulted in the victim being labeled as promiscuous and to blame for having produced such images in the first place. This was primarily an issue for girls. In a more recent review of outcomes of sexting, Doyle et al. (2021) looked at 54 papers published between 2012 and 2021 and found four overarching categories of outcomes: psychological, behavioral, relational, and system-level. Similarly to Anastassiou, Doyle et al. (2021) found several positive outcomes from sexting connected to quality of life, emotional outcomes, and relational and reputational outcomes. Such positive outcomes were generally associated with consensual and respectful sexting behavior. At the same time, Doyle et al. (2021) also found several negative outcomes regarding mental health and quality of life. Doyle et al. found behavioral outcomes, hereunder perpetration of abuse and harassment, and sexual activity and risk behaviors—with the latter referring to risky sexual behavior, such as unprotected sex and multiple concurrent sexual partners. Furthermore, they found relational outcomes, consisting of difficulties in personal connections with others and reputational outcomes. Reputational outcomes appeared to be gender-dependent, as boys more frequently reported positive reputational outcomes and girls reported the opposite. Lastly, systems-level outcomes included worry about, and negative outcomes from, distribution/public exposure of sexting content. Finally, Dodaj et al. (2022) reviewed 11 mixed methods studies, published between 2014 and 2022. Similarly to Anastassiou (2017) and Doyle et al. (2021), they found that studies tended to report both positive and negative outcomes from sexting. Positive outcomes included sexual arousal, stimulation, flirting, validation, mutual trust, entertainment and beneficial for intimate and sexual relationships. Negative outcomes included shame, guilt, breakdown in relationships, loss of control, embarrassment, humiliation, and reputational damage.

Findings thereby show that sexting can have both positive and negative outcomes for those involved. However, negative outcomes are arguably more related to the exploitation of sexting content in the facilitation of abusive and violent behavior, rather than sexting in itself (Krieger, 2017). This is supported by Dodaj et al. (2022), who argue that the consequences of sexting can be understood through a framework of sexting from a normal and a deviant perspective. Here positive outcomes are associated with relational and reactive sexting, while negative consequences are associated with forced and abusive sexting.

### 2.1 Rationale, aims, and research questions

Existing knowledge on image-based sexual abuse—largely derived from research on sexting—reveals a troubling pattern of harm, particularly for young people. However, while the prevalence of IBSA is increasingly recognized, less is known about the specific consequences for victims. This systematic review aims to synthesize existing empirical research on the outcomes of IBSA among individuals aged 10–24. By doing so, it seeks to inform clinical, legal, and policy responses to this form of abuse. The review addresses the following questions:

- What outcomes are reported by young victims of IBSA?
- What are the implications for legal and institutional responses?

In answering these questions, the review aims to contribute to a more evidence-based and trauma-informed understanding of how IBSA affects young people and how institutions can respond more effectively.

## 3 Methods

**Figure 1:**
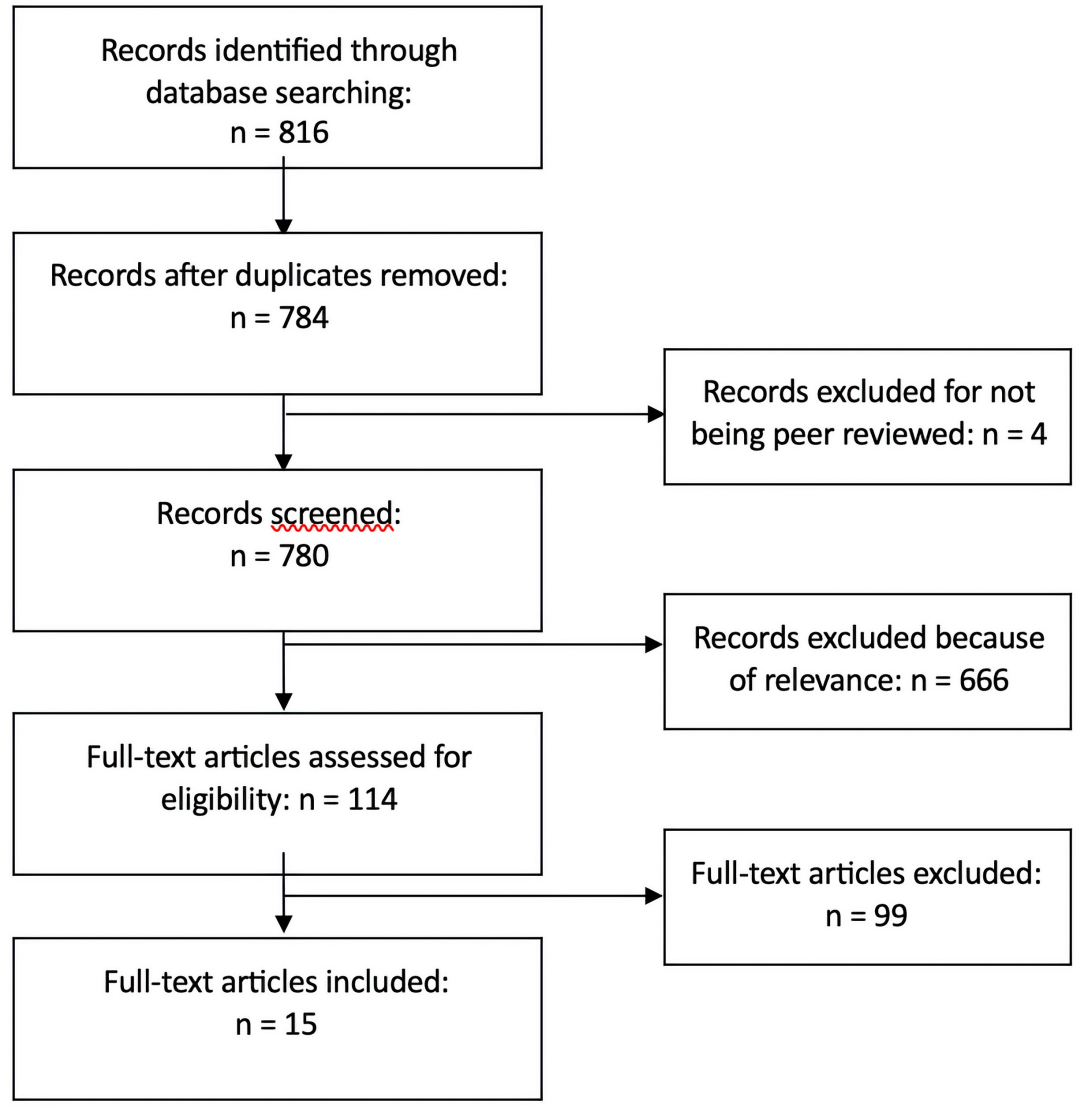
Flow diagram.

This systematic review follows the PRISMA (Preferred Reporting Items for Systematic Reviews and Meta-Analyses) guidelines for producing systematic reviews in research (Moher et al., 2009). The following criteria were used to include identified studies: (1) Original empirical data, (2) Outcomes of IBSA, (3) English-language journals, (4) Age 10–24.^1^ We omitted articles by the following exclusion criteria: (1) Non-English language journals, (2) Not original empirical data, (3) Not reporting on outcomes, (4) Participants younger than 10 or older than 24 years old only. See Figure 1 for PRISMA flow diagram.

We underscore here that papers had to specifically address outcomes of IBSA. This means that studies that described outcomes from sexting in general, without specifying the abusive behavior (e.g., unwanted distribution of intimate images), were not included. However, papers that looked at sexting, but also detailed the underlying abusive behavior that resulted in specific outcomes, were included.

### 3.1 Literature search

The second author, in cooperation with a librarian with expertise in systematic review methodology, performed the literature searches. The searches, using the databases PsycInfo, Sociological Abstracts and Google Scholar, were performed in June 2024. We restricted our search to articles published between 2010 and 2024, and the included articles are from 2017 to 2022. All searches were documented in detail. The following terms were included in the searches: “image-based sexual abuse, nudes, sexting, “self-produced sexual images,” “sexual images,” “revenge porn,” “sexual videos,” adolescent^*^, teen^*^, young, “young adults,” “young people,” youth, girl, boy, impact, outcome, consequence^*^, result, effect, reaction, “mental health,” anxiety, depression, psychology. Additionally, the following terms were identified throughout the search process and included in the subsequent searches: online sexual offenses, non-consensually disseminated sexually explicit media (NCDSEM), online sexual harassment, sexual online victimization, technology-assisted sexual abuse, electronic image-sharing, sextortion, online sexual engagement, cybervictimization, technology-facilitated sexual violence, youth digital sexual image exchange, “frexting,” online sexual experiences, nonconsensual sharing of private sexually explicit media, sexting coercion, online sexual victimization and risks (OSVR).

### 3.2 Screening of data

We identified 89 articles eligible for full-text assessment through the searches, and similarly 15 articles through Google Scholar. An additional 11 articles were identified through screening reference list of articles. By removing one duplicate, a total of 114 articles were screened by three researchers independently and a total of 15 articles were seen as relevant for inclusion in the review. The researchers then reviewed the articles and categorized them based on content and research questions. After thorough analysis and discussion, we reached a final consensus regarding the categorization of the articles.

### 3.3 Quality assessment of research papers

The quality of the 15 articles was then assessed using the “Standard quality assessment criteria for evaluating primary research papers from a variety of fields” (Kmet et al., 2004). This provides guidelines for assessing the quality of research papers in various fields, through assessing aspects such as description of research objective(s), study design, connection to a theoretical framework and more. The score for each article is the total sum divided by the total possible sum and can range from 0 to 1. As the topic of specific outcomes of IBSA is relatively little explored in the research literature, we decided on a relatively liberal cut-point of 0.50 for article inclusion. Of the 15 papers that were assessed for quality, 12 fulfilled the agreed upon level for inclusion (see Table 1).

**Table 1:** Quality assessment.

**Paper**	**Point 1**	**Point 2**	**Point 3**	**Point 4**	**Point 5**	**Point 6**	**Point 7**	**Point 8**	**Point 9**	**Point 10**	**Total**	**Score**
Campbell, J. K., Poage, S. M., Godley, S., & Rothman, E. F. (2022). Social anxiety as a Consequence of Non-consensually Disseminated Sexually Explicit Media Victimization.	2	2	1	1	1	2	2	0	2	0	13	0.65
Champion, A. R., Oswald, F., Khera, D., & Pedersen, C. L. (2022). Examining the gendered impacts of technology–facilitated sexual violence: A mixed method approach	2	2	1	2	2	2	1	2	2	0	16	0.8
Harder, S. K. (2021). The emotional bystander – sexting and image–based sexual abuse among young adults	1	2	2	2	2	2	1	0	2	0	14	0.7
Mandau, M. B. H. (2020). ‘Directly in Your Face': A Qualitative Study on the Sending and Receiving of Unsolicited ‘Dick Pics' Among Young Adults.	1	2	2	2	2	2	1	0	2	0	14	0.7
Mandau, M. B. H. (2021). “Snaps”, “screenshots”, and self–blame: A qualitative study of image–based sexual abuse victimization among adolescent Danish girls. Journal of Children and Media, 15(3), 431–447	2	1	2	2	1	1	2	0	2	0	13	0.65
Meehan, C. (2021). ‘It's like Mental Rape I Guess': Young New Zealanders' Responses to Image–Based Sexual Abuse. In (pp. 281–295)	1	2	2	2	2	1	2	0	2	1	15	0.75
Meehan, C. (2022). ‘If someone's freaky, everyone loves it. It's all about the drama': young women's responses and reactions to image based sexual abuse of other young women.	1	2	1	2	1	2	1	0	2	2	14	0.7
Ringrose, J., Milne, B., Mishna, F., Regehr, K., & Slane, A. (2022). Young people's experiences of image–based sexual harassment and abuse in England and Canada: Toward a feminist framing of technologically facilitated sexual violence	1	1	2	2	1	1	1	0	2	0	11	0.55
Ringrose, J., Regehr, K. & Whitehead, S. (2022) ‘Wanna trade?': Cisheteronormative homosocial masculinity and the normalization of abuse in youth digital sexual image exchange, Journal of Gender Studies, 31:2, 243–261. doi: 10.1080/09589236.2021.1947206	1	2	2	2	1	1	1	0	2	0	12	0.6
Setty, E. (2019). Meanings of Bodily and Sexual Expression in Youth Sexting Culture: Young Women's Negotiation of Gendered Risks and Harms. Sex Roles, 80(9), 586–606.	2	2	2	2	1	2	2	0	2	2	17	0.85
Van Ouytsel, J., Van Gool, E., Walrave, M., Ponnet, K., & Peeters, E. (2017). Sexting: adolescents' perceptions of the applications used for, motives for, and consequences of sexting	2	1	2	2	1	2	2	0	2	0	14	0.7
Wolak, J., Finkelhor, D., Walsh, W., & Treitman, L. (2018). Sextortion of Minors: Characteristics and Dynamics. J Adolesc Health, 62(1), 72–79	2	2	2	2	1	2	2	0	2	2	17	0.85

^*^Standard Quality Assessment Criteria for Evaluating Primary Research Papers (Kmet et al., 2004).

## 4 Results

The 12 papers that met the requirements to be included in this review were published between 2017 and 2022, with ten of them being qualitative interview studies where the respondents themselves attributed outcomes to their experiences with IBSA, one quantitative study, and one mixed methods study (see Table 2). After analyzing the studies, three overarching categories were established: (1) psychological and emotional outcomes; (2) social outcomes; and (3) educational and occupational outcomes.

**Table 2:** Included papers.

**Author/year**	**Country**	**Design**	**Recruitment setting**	**Sampling**	***N***	**Sample age**	**Outcomes**
Campbell et al. (2022)	USA	Qualitative	NGO^*^, social media	Self-selection	17	21–22	*Psychological/emotional:* Fear, worry, depression, increased social anxiety, isolation, loss of trust, feelings of worthlessness, overthinking/overanalyze, broken, sad, stressed, anxious, PTSD, panic attack that required hospitalization
Champion et al. (2022)	Canada/USA	Mixed methods	Social media	Criterion sampling	Survey = 337Interviews = 10	14–23	*Psychological/emotional:* Depressed, terrified, lack of control, fear of future victimization, embarrassment, severe anxiety, severe paranoia, bullied*Social:* Bullied, (sexually) harassed, more sexual dominant (for protection)*Educational/occupational:* Lower occupational functioning, quit job and relocate
Harder (2021)	Denmark	Qualitative	Dating app, schools	Self-selection	25	18–24	*Psychological/emotional:* Guilt, awkwardness, shame, stomachache, laughing it off (dickpics), shock, upset
Mandau (2020)	Denmark	Qualitative	Schools			17–20	Psychological/emotional: Shock, laughing it off (dickpics)
Mandau (2021)	Denmark	Qualitative	Online posts	Textual sampling	157 posts	11–20	Psychological/emotional: Self-blame, fear and worry, sadness, suicidal thoughts, anger, loss of self-worth, disappointment, regret
Meehan (2021)	New Zealand	Qualitative	Schools	Self-selection	106	12–16	Psychological/emotional: Feeling disgusting/dirty/used, shame *Educational/occupational:* move schools
Meehan (2022)	New Zealand	Qualitative	Schools	Self-selection	106	12–16	*Psychological/emotional:* emotional harm, humiliation, distress, anxiety*Social:* ostracized from friendship groups, bullied, punished, (sexually) harassed (slut)shamed, blamed*Educational/occupational:* Move schools
Ringrose et al. (2022a)	United Kingdom/Canada	Qualitative	Schools	Self-selection	206	11–19	*Social:* Shamed, praised (boys for sending dick pics)
Ringrose et al. (2022b)	United Kingdom/Canada	Qualitative	Schools	Self-selection	144	11–18	*Social:* Reputationally damaged, shamed, ostracized from friendship groups, lightheartedly teased (boy)
Setty (2019)	United Kingdom	Qualitative	Schools, youth clubs	Self-selection	41	14–18	Psychological/emotional: Shocked, upset, self-blame, shame, feelings of unpleasantness, offended*Social:* Bullied
Van Ouytsel et al. (2017)	Belgium	Qualitative	Schools	Self-selection	57	15–18	*Social:* (Sexually) harassed, blamed, bullied
Wolak et al. (2018)	USA	Quantitative	Social media	Self-selection	572	13–17	*Psychological/emotional:* Saw mental health or medical practitioner*Social:* Lost relationship with friend or family member *Educational/occupational:* left or changed school or had school-related problem; moved to new neighborhood, community, or town; incurred financial costs; left or changed job or had job-related problem

The age ranges reflect the ranges that are included in this review. However, the actual age ranges are greater in some of the studies.

^*^The NGO Battling Abusive and Demeaning Selfie Sharing (BADASS).

### 4.1 Psychological and emotional outcomes

This category includes the negative effects of IBSA victimization on psychological and emotional wellbeing, ranging from feelings of unpleasantness to post traumatic stress disorder and even suicidal thoughts (see Table 3). The most frequently reported forms of psychological and emotional outcomes were shock and fear (Campbell et al., 2022; Champion et al., 2022; Harder, 2021; Mandau, 2020, 2021; Setty, 2019). This includes both the initial shock of being the victim of IBSA, and also a fear of future victimization and being anxious of what will happen. Another frequently reported psychological and emotional outcome was shame (Harder, 2021; Meehan, 2021; Setty, 2019; Van Ouytsel et al., 2017). Interestingly, Harder (2021) reported shame in bystanders who were exposed to the non-consensual sharing of intimate images. Such shame stemmed from the bystander feeling that they did not react in accordance with their morals in the situation, and rather than calling out the perpetrator for their behavior they would resort to not doing anything. Noteworthy, one of the respondents in this study argued against feelings of shame related to her sexting, rather framing the sending of intimate images as normal, sexual behavior. In her view, the shame should be put on persons who distribute such images without consent. However, the opposite appears to be common, as feelings of self-blame were reported in two of the studies (Mandau, 2021; Setty, 2019), and guilt, as reported by Harder (2021). Moreover, two studies reported victims feeling worthless/a loss of self-worth as an outcome of IBSA (Campbell et al., 2022; Mandau, 2021).

**Table 3:** Psychological and emotional outcomes.

**Grouped outcomes**	**Study/studies**	**Description/details**
**Most frequently reported outcomes**		
Shock and fear	Campbell et al. (2022), Champion et al. (2022), Harder (2021), Mandau (2020, 2021), Setty (2019)	Initial shock and fear of future victimization or exposure
Shame	Harder (2021), Meehan (2021), Setty (2019), Van Ouytsel et al. (2017)	Feelings of shame, including among bystanders; often gendered and socially imposed
**Other outcomes**		
Guilt, self-blame, loss of self-worth	Harder (2021), Mandau (2021), Setty (2019), Campbell et al. (2022)	Feelings of responsibility, diminished self-esteem, or moral conflict
Worry, stress/distress, upset, sadness	Campbell et al. (2022), Mandau (2021), Meehan (2022), Harder (2021), Setty (2019)	Common emotional discomforts following victimization
Depression and anxiety	Campbell et al. (2022), Champion et al. (2022), Meehan (2022)	Symptoms of mood and anxiety disorders
Laughter (as coping or reaction)	Harder (2021), Mandau (2020)	Reported in response to unsolicited explicit images, particularly “dick pics”
Broader psychological/ emotional harm	Mandau (2021), Setty (2019), Meehan (2021, 2022), Champion et al. (2022), Campbell et al. (2022), Wolak et al. (2018)	Includes anger, feeling offended, disappointment, embarrassment, awkwardness, emotional harm, humiliation, unpleasantness, regret, loss of control, overthinking, feeling broken, social anxiety, severe paranoia, and seeking mental/medical help
**More severe outcomes**		
PTSD and panic attacks	Campbell et al. (2022)	Clinical trauma symptoms resulting from IBSA
Suicidal thoughts	Mandau (2021)	Reports of suicidal ideation among victims

Other reported reactions included worry (Campbell et al., 2022; Mandau, 2021), distress or stress (Campbell et al., 2022; Meehan, 2022), feeling upset (Harder, 2021; Setty, 2019), and sadness (Campbell et al., 2022; Mandau, 2021), each identified in two studies. Depression (Campbell et al., 2022; Champion et al., 2022) and anxiety (Champion et al., 2022; Meehan, 2022) were also each reported by two studies. Two additional studies found that some respondents laughed at the situation; notably, both focused on reactions to unsolicited “dick pics” (Harder, 2021; Mandau, 2020). Harder (2021) examined the responses of bystanders who witnessed IBSA, while Mandau (2020) explored girls' reactions to receiving such images.

A wide range of other emotional responses were also documented, including anger (Mandau, 2021), feeling offended (Setty, 2019), shame or feeling dirty and used (Meehan, 2021), disappointment (Mandau, 2021), embarrassment (Champion et al., 2022), awkwardness (Harder, 2021), emotional harm and humiliation (Meehan, 2022), unpleasantness (Setty, 2019), regret (Mandau, 2021), loss of control (Mandau, 2021), overthinking (Campbell et al., 2022), feeling broken (Campbell et al., 2022), social anxiety (Campbell et al., 2022), and severe paranoia (Champion et al., 2022). Contacting mental health or medical professionals was also reported as a consequence in some cases (Wolak et al., 2018).

Finally, more severe psychological outcomes were observed. Campbell et al. (2022) reported instances of post-traumatic stress disorder (PTSD) and panic attacks, while Mandau (2021) identified cases of suicidal ideation. Taken together, these findings underscore the broad and potentially severe psychological and emotional impact of IBSA on victims.

### 4.2 Social outcomes

IBSA can also have significant social consequences, many by actions from the victim's social surroundings, but also outcomes shaped by the victim's coping responses—such as social withdrawal or loss of trust (see Table 4). The most common social outcome was being bullied, with a total of four studies reporting this (Champion et al., 2022; Meehan, 2022; Setty, 2019; Van Ouytsel et al., 2017). Meehan (2022) highlights how bullying functions as a tool for social sanctioning and control, signaling what is regarded as sexually permissible by holding victims of IBSA responsible for their own victimization. Similarly, Setty (2019) describes how bullying resulted in a victim of IBSA “*reappraising her experience and constructing it as her ‘mistake'*” (p. 32). A related social outcome was harassment, including sexual harassment, reported by three studies (Champion et al., 2022; Meehan, 2022; Van Ouytsel et al., 2017). Similarly to bullying, (sexual) harassment appears to revolve around sanctioning and controlling (mainly) women's sexuality (Meehan, 2022). Interestingly, Van Ouytsel et al. (2017) reported that in some instances, peer groups would quickly move on from incidents of non-consensual distribution of intimate images, without socially sanctioning the victim. Van Ouytsel et al. (2017) argue that a potential explanation for this could be a normalization of sexting, as this behavior is relatively prevalent among young people, where having images distributed without one's consent is becoming less out of the norm. However, as with bullying and harassment, shaming and blaming of victims was another common social outcome, with three studies reporting shaming and two reporting blaming (Meehan, 2022; Ringrose et al., 2022a,b; Van Ouytsel et al., 2017). Similarly to other social outcomes, girls appear to be more targeted than boys (Ringrose et al., 2022b). Ringrose et al. (2022b) reported that boys would actively use social media to shame girls who had sent intimate images. In comparison, boys who had sent intimate images of themselves seldomly faced any social sanctions because of their actions. Moreover, in instances where boys had redistributed intimate images without consent, blame was frequently put on the victim rather than the boy spreading the images (Meehan, 2022). Furthermore, both Ringrose et al. (2022b) and Ringrose et al. (2022a) reported that boys sending dick pics, even if they were re-distributed without consent, were met with lighthearted banter and praise, rather than the negative social sanctions directed at girls.

**Table 4:** Social outcomes.

**Outcome**	**Study/studies**	**Description/details**
Bullying	Champion et al. (2022), Meehan (2022), Setty (2019), Van Ouytsel et al. (2017)	Victims were bullied, often as a form of social sanctioning, reinforcing gendered norms around sexuality
Harassment (including sexual harassment)	Champion et al. (2022), Meehan (2022), Van Ouytsel et al. (2017)	Victims experienced harassment aimed at controlling or punishing perceived transgressions of sexual norms
Shaming	Meehan (2022), Ringrose et al. (2022a), Ringrose et al. (2022b)	Victims, especially girls, were publicly shamed, often via social media, reinforcing gender inequality
Victim-blaming	Meehan (2022), Van Ouytsel et al. (2017)	Victims were blamed for the abuse they experienced, rather than those who distributed the images
Ostracization	Meehan (2022), Ringrose et al. (2022b)	Victims were excluded from peer or friendship groups following the abuse
Loss of relationships	Wolak et al. (2018)	Victims reported losing friendships or familial ties as a consequence of the abuse
Punishment	Meehan (2022)	Some victims faced disciplinary measures or punishment following the incident
Reputational damage	Ringrose et al. (2022b)	Victims' reputations were negatively affected, especially in school or online environments
Loss of trust	Campbell et al. (2022)	Victims described a general loss of trust in others and strained interpersonal relationships

Social outcomes of IBSA also include being ostracized from friendship groups (Meehan, 2022; Ringrose et al., 2022b) and losing relationships with friends and/or family members (Wolak et al., 2018). Social outcomes also included being punished (Meehan, 2022) and reputational damage (Ringrose et al., 2022b). As mentioned, social outcomes were sometimes shaped by the victim's coping responses. For example, Campbell et al. (2022) found that victims reported a general loss of trust toward people. Fear of, and actual, negative social reactions affected victims' relationships with others, both new relationships and existing ones, including online (Campbell et al., 2022).

### 4.3 Educational and occupational outcomes

Finally, outcomes to a victim's educational and occupational circumstances were frequent in the included papers. Such outcomes include having to move schools because of IBSA (Meehan, 2021, 2022; Wolak et al., 2018), having to quit a job or having lower occupational functioning (Champion et al., 2022), having to move to a new neighborhood (Champion et al., 2022), or incurred financial costs (Wolak et al., 2018) (see Table 5). The most common educational and occupational outcome was having to move school, with three studies reporting this outcome (Meehan, 2021, 2022; Wolak et al., 2018). Similarly, Champion et al. (2022) found that victims of IBSA had to quit their job and relocate because of severe embarrassment. Not surprisingly, Champion et al. (2022) also found that IBSA victimization could have negative effects on a person's occupational functioning. Furthermore, Wolak et al. (2018) found that as many as one in 10 of IBSA victims aged 17 or younger at the time of the victimization, moved to a new neighborhood/community/or town because of the abuse. These studies clearly show that IBSA victimization not only affects victims' psychological and social wellbeing, but also their specific educational and occupational situations, further increasing the burden and potential harm to young people already in a highly difficult situation.

**Table 5:** Educational and occupational outcomes.

**Outcome**	**Study/studies**	**Description/details**
School relocation	Meehan (2021), Meehan (2022), Wolak et al. (2018)	Victims had to change schools due to stigma, bullying, or safety concerns
Job loss/quitting employment	Champion et al. (2022)	Victims left employment due to shame or reputational harm
Reduced occupational functioning	Champion et al. (2022)	Reported decline in job performance and ability to maintain work-related responsibilities
Residential relocation	Champion et al. (2022), Wolak et al. (2018)	Victims and/or families moved to a new neighborhood, community, or town
Financial costs	Wolak et al. (2018)	Victimization led to economic burdens (e.g., legal fees, therapy, moving expenses)

## 5 Discussion

This review demonstrates that image-based sexual abuse (IBSA) has substantial and multifaceted consequences for young people's psychological, social, and academic lives. Victims report a range of outcomes, including anxiety, depression, shame, social withdrawal, school disruption, and job loss, many of which align with clinical thresholds for psychological distress (Campbell et al., 2022; Champion et al., 2022). The compounding effects of social exclusion and institutional responses—such as victim-blaming and school relocation—further exacerbate these harms and may lead to long-term disengagement from social settings and education (Meehan, 2022; Wolak et al., 2018).

When contextualized within the broader literature on sexual abuse, these findings reveal notable parallels between the impacts of IBSA and those of physical sexual abuse. Prior research has shown that victims of offline sexual abuse frequently experience long-term consequences, including PTSD, suicidality, and educational and occupational disruption (Browne and Finkelhor, 1986; Maniglio, 2009). Similar long-term effects have also been observed among adult victims of digital sexual violence, including image-based abuse (Henry et al., 2020, 2022; Huber, 2023). The digital dimension of IBSA introduces unique factors—such as the permanence and replicability of images, as well as their potential for mass dissemination—which may intensify trauma and complicate recovery (Huber, 2020; Meechan-Rogers et al., 2021). This review further highlights that outcomes of IBSA are not limited to psychological harm. Victims also experience reputational damage, exclusion from peer groups, and reduced trust in others, all of which can significantly impair social development and belonging (Champion et al., 2022; Ringrose et al., 2022a). These findings align with research on the psychosocial impacts of adolescent victimization more broadly, which has shown that abuse during formative years can undermine autonomy, identity formation, and interpersonal trust (Crone and Dahl, 2012; Tolman and McClelland, 2011).

The overlap between IBSA and physical sexual abuse also extends to educational and occupational consequences. Victims of IBSA in several studies reported school relocation, impaired academic engagement, and job loss (Meehan, 2021; Champion et al., 2022). These outcomes mirror findings from studies on childhood sexual abuse, which demonstrate associations with school dropout, reduced academic performance, and long-term economic disadvantage (Henkhaus, 2022; McLaughlin et al., 2017; Mitchell et al., 2021). That school transfers themselves are associated with increased dropout risk further highlights the compounding nature of IBSA-related disruptions (Gasper et al., 2012). Moreover, this review draws attention to the role of peer and institutional responses in reinforcing harm. Victim-blaming, shaming, and gendered double standards were consistently reported, reflecting broader societal narratives that excuse male behavior while stigmatizing girls (Flynn et al., 2023; Ringrose et al., 2022b). Such dynamics are well-documented in both digital and physical contexts and are known to contribute to secondary victimization (Gravelin et al., 2019; Anderson and Overby, 2021).

It is also essential that professionals acknowledge the severity of IBSA and recognize that its consequences can be as profound and far-reaching as those associated with physical sexual abuse. These insights should guide ongoing efforts to refine theoretical frameworks and empirical tools for understanding the unique dynamics and long-term harms associated with digital sexual victimization. In particular, forensic psychological practice must be equipped to recognize and evaluate the psychological impacts of IBSA, especially in young people who may not voluntarily disclose their experiences. Assessments should consider the digital permanence, reputational fallout, and social isolation that often accompany IBSA. Legal frameworks should likewise evolve to reflect the seriousness of such harms, moving beyond traditional conceptions of sexual abuse to account for the specific mechanisms and consequences of digital violations. A deeper integration of forensic psychological insight into legal and policy discourse is essential to ensure that victims of IBSA receive adequate recognition, support, and justice.

### 5.1 Strengths and limitations

This literature review synthesizes findings from 12 studies on the outcomes of image-based sexual abuse (IBSA), providing a comprehensive understanding of its impact on individuals aged 10 to 24 in Western contexts. The studies analyzed include one mixed-methods study, one quantitative study, and 10 qualitative studies, covering diverse methodological approaches and enabling triangulation of findings. A significant strength of this review lies in its qualitative focus, which enables an in-depth exploration of victims' lived experiences and the complexities of their emotional, social, and situational challenges. This emphasis offers valuable insights into the personal and contextual realities of IBSA, which are often difficult to capture through purely quantitative methods. Additionally, the focus on a critical developmental age group—spanning adolescence and early adulthood—enhances the relevance of the findings for understanding how IBSA affects individuals during periods of heightened vulnerability and identity formation. At the same time, the predominance of qualitative studies presents a limitation in terms of generalizability. Additionally, while the categorization of outcomes provides clarity, it may oversimplify the interplay between domains, such as how emotional distress influences social relationships or academic achievements. Finally, the time range of the included studies, while recent, may not fully account for the rapidly changing digital landscape, particularly the emergence of new technologies and platforms that facilitate IBSA. Consequently, the findings may not fully reflect the experiences of individuals exposed to the most recent developments in digital media. Additionally, while this review's focus on lived experiences across multiple jurisdictions provides a foundation for comparative legal-psychological reflection, the included studies are all from Western contexts. Thereby, further cross-cultural research is needed to inform legal practice globally.

### 5.2 Implications for practice, policy and research

The findings of this review underscore several important implications for forensic psychological practice, legal policy, and future research. First, the range and severity of emotional and psychological outcomes—including anxiety, depression, and post-traumatic symptoms—highlight the need for routine assessment of digital sexual victimization in forensic evaluations of young people. These assessments should also inform judicial decision-making regarding both victims and perpetrators in cases involving technology-facilitated sexual violence. Second, the widespread victim-blaming and reputational harm observed—particularly among girls—suggest that legal professionals, educators, and social service providers must be trained in gender-sensitive, trauma-informed approaches to reduce further victimization. This includes targeted prevention strategies and awareness campaigns, as well as institutional procedures that acknowledge the specific harm caused by IBSA. Third, findings showing disruptions in schooling, employment, and social relationships indicate that IBSA has far-reaching implications beyond the digital sphere. These consequences should be considered in legal actions, school-based interventions, and social policy development. Finally, the systematic gaps in research—especially longitudinal studies and non-Western perspectives—call for an expanded empirical agenda. Future studies should examine protective factors, enhancement of prevention efforts, and the effectiveness of legal and therapeutic responses to IBSA, with particular attention to adolescents' digital vulnerability and rights within legal systems.

## 6 Conclusion

Image-based sexual abuse is a serious and harmful form of digital victimization with significant negative psychological, social, and physiological outcomes for victims. Similarities between outcomes of IBSA and outcomes of face-to-face sexual abuse highlight the severity of digital sexual violence and abuse and the importance of recognizing that digital victimization can be as harmful to those involved as “traditional” victimization. At the same time, when working to prevent IBSA and other forms of digital sexual violence and abuse and when providing help to those involved, the unique aspects of digital victimization and how they can affect its outcomes must also be recognized. Unfortunately, as with sexual violence and abuse in general, the tendency to blame the victim can exacerbate negative outcomes. In conclusion, image-based sexual abuse among young people presents a serious threat to psychological wellbeing and social inclusion, with clear implications for forensic and legal psychology. The findings from the reviewed studies underscore the need for a trauma-informed, legally responsive framework that acknowledges the severity of digital sexual abuse. Psychological assessment, victim support, and legal decision-making must reflect the complex harm inflicted by IBSA. Addressing this form of abuse supports not only individual recovery but also broader societal goals related to gender equality, justice, and the protection of vulnerable young people.

## Data Availability

The original contributions presented in the study are included in the article/supplementary material, further inquiries can be directed to the corresponding author.

## Footnotes

^1^In accordance with the United Nations Department of Economic and Social Affairs' (UNDESA) definition of young people (UNDESA, 2013).
